# Tissue distribution and acute toxicity of silver after single intravenous administration in mice: nano-specific and size-dependent effects

**DOI:** 10.1186/s12989-016-0124-x

**Published:** 2016-02-29

**Authors:** Camilla Recordati, Marcella De Maglie, Silvia Bianchessi, Simona Argentiere, Claudia Cella, Silvana Mattiello, Francesco Cubadda, Federica Aureli, Marilena D’Amato, Andrea Raggi, Cristina Lenardi, Paolo Milani, Eugenio Scanziani

**Affiliations:** 1 Fondazione Filarete, 20139 Milan, Italy; 2 Dipartimento di Scienze Veterinarie e Sanità Pubblica (DIVET), Università degli Studi di Milano, 20133 Milan, Italy; 3 Dipartimento di Fisica, Università degli Studi di Milano, 20133 Milan, Italy; 4 Department of Food Safety and Veterinary Public Health, Istituto Superiore di Sanità - National Health Institute, 00161 Rome, Italy; 5 Centro Interdisciplinare Materiali e Interfacce Nanostrutturati (CIMAINA), Università degli Studi di Milano, 20133 Milan, Italy

**Keywords:** Silver nanoparticles, Silver acetate, Dissolution, *In vivo* study, Mouse, Intravenous route, Tissue distribution, Toxicity, Hepatocellular necrosis, Hemorrhage

## Abstract

**Background:**

Silver nanoparticles (AgNPs) are an important class of nanomaterials used as antimicrobial agents for a wide range of medical and industrial applications. However toxicity of AgNPs and impact of their physicochemical characteristics in *in vivo* models still need to be comprehensively characterized. The aim of this study was to investigate the effect of size and coating on tissue distribution and toxicity of AgNPs after intravenous administration in mice, and compare the results with those obtained after silver acetate administration.

**Methods:**

Male CD-1(ICR) mice were intravenously injected with AgNPs of different sizes (10 nm, 40 nm, 100 nm), citrate-or polyvinylpyrrolidone-coated, at a single dose of 10 mg/kg bw. An equivalent dose of silver ions was administered as silver acetate. Mice were euthanized 24 h after the treatment, and silver quantification by ICP-MS and histopathology were performed on spleen, liver, lungs, kidneys, brain, and blood.

**Results:**

For all particle sizes, regardless of their coating, the highest silver concentrations were found in the spleen and liver, followed by lung, kidney, and brain. Silver concentrations were significantly higher in the spleen, lung, kidney, brain, and blood of mice treated with 10 nm AgNPs than those treated with larger particles. Relevant toxic effects (midzonal hepatocellular necrosis, gall bladder hemorrhage) were found in mice treated with 10 nm AgNPs, while in mice treated with 40 nm and 100 nm AgNPs lesions were milder or negligible, respectively. In mice treated with silver acetate, silver concentrations were significantly lower in the spleen and lung, and higher in the kidney than in mice treated with 10 nm AgNPs, and a different target organ of toxicity was identified (kidney).

**Conclusions:**

Administration of the smallest (10 nm) nanoparticles resulted in enhanced silver tissue distribution and overt hepatobiliary toxicity compared to larger ones (40 and 100 nm), while coating had no relevant impact. Distinct patterns of tissue distribution and toxicity were observed after silver acetate administration. It is concluded that if AgNPs become systemically available, they behave differently from ionic silver, exerting distinct and size-dependent effects, strictly related to the nanoparticulate form.

**Electronic supplementary material:**

The online version of this article (doi:10.1186/s12989-016-0124-x) contains supplementary material, which is available to authorized users.

## Background

Silver nanoparticles (AgNPs) are an important class of nanomaterials characterized by sizes ranging approximately from 1 to 100 nm: these small dimensions result in a high surface area to volume ratio determining unique chemical, physical and biological properties different from those of bulk material with the same composition [1]. Nowadays, AgNPs are the most common nanomaterial found in consumer products (including cosmetics, textiles, food boxes, sprays), appliances (refrigerators, washing machines) and medical applications (wound dressings, medical devices, drug-delivery systems, bio-sensing and imaging methods) [2–5]. The widespread application of AgNPs is mainly related to the renowned antimicrobial activity of silver, whether ionic or nanoparticulate [6, 7]. However, extensive use of AgNPs may lead to environmental contamination and human exposure by inhalation, dermal and oral routes, raising concerns about their potential environmental impact and toxicity [4].

The majority of toxicity studies on AgNPs have been performed on bacteria, cell lines, and non-mammalian animal species, with still comparatively limited information available from *in vivo* studies [1, 8]. *In vitro* studies revealed distinct (but not necessarily mutually exclusive) mechanisms of toxicity of AgNPs, including 1) ROS generation, with subsequent oxidative stress; 2) interaction with cellular proteins and enzymes by binding to free thiol groups; and 3) mimicry of endogenous ions (e.g. calcium, sodium, or potassium) leading to ionoregulatory disturbances [1]. These mechanisms lead to cytokine production, cellular damage, and eventually apoptosis or necrosis. Numerous *in vitro* studies have demonstrated that the cytotoxic and genotoxic effects of AgNPs are size- and dose-dependent, as well as coating- and cell type-dependent [9–14].

*In vivo* studies in rodents (rats, mice, guinea pigs) and occasionally in non-rodents species (pigs) have been carried out, using different routes of exposure, in the attempt to characterize kinetics, tissue distribution and toxicity of AgNPs [15–26]. Compared to the general consensus of *in vitro* studies, the results of the *in vivo* studies are controversial regarding the onset of adverse effects after AgNPs administration. Some of these studies indicate that AgNPs may have toxic effects on liver, lung, intestine, nervous and immune systems, either after single or repeated administration, and following different routes of exposure [15–17, 22, 23, 26]. However, other studies found no relevant adverse effects [19–21, 24]. These contradictory results may depend on the high variability of the tested AgNPs, in terms of source (generated in the laboratory or commercially available materials), size, dispersion state, coating, and concentration (i.e. number of particles and silver mass). Also, the animal species, strain, sex, age, and the overall experimental design (dose, exposure time, end points for sampling) may have an impact on the outcome of the study [21].

With the effort to standardize and compare *in vivo* experiments, as well as to properly correlate nanoparticles’ properties with their *in vivo* effects, a prior and rigorous physicochemical characterization of AgNPs is required [27]. In particular, the assessment of size, monodispersity, and aggregation is fundamental for a comprehensive understanding of their biological effects. However such measurements, albeit fundamental, are not sufficient for fully predicting nanotoxicological effects, likely because of effects related to the still poorly understood behavior of nanoparticles in the biological milieu. Indeed, AgNPs after injection readily interact with blood components (proteins, lipids, etc.), forming on their surface a biomolecular corona that influences the biological interactions of nanoparticles and cellular uptake [28]. *In vivo* toxicological studies are thus considered critical for correlating the physicochemical properties of nanoparticles with their effects in living systems [29]. However, only very few *in vivo* studies have been performed so far to evaluate the role of size and coating of AgNPs, and based on these studies tissue distribution and toxicity appeared to be consistently size-dependent, whereas the effect of coating was less obvious and only observed in the lung [21, 30–33]. In addition to influencing the formation of the biomolecular corona, size and coating are critical factors affecting the release of silver ions from AgNPs. There has been much debate in the literature regarding whether the adverse effects caused by AgNPs are mediated by the release of silver ions [34]. Although it is generally accepted that dissolution of AgNPs does account for at least a degree of toxicity observed under AgNP exposure, it appears that effects cannot be fully attributed to the measured dissolved fraction of silver especially for the smaller particles (≤10 nm), which proved to be more toxic than predicted on the basis of silver ion release [35, 36].

In view of the need to improve the understanding of the impact of physicochemical characteristics of AgNPs in *in vivo* models, the aim of this study was to investigate the effect of the size and coating on tissue distribution and toxicity of AgNPs and compare the results with those obtained after administration of silver ions, in the form of silver acetate. Since this study was not intended to mimic human exposure scenarios, to avoid limited systemic exposure due to the cellular barriers present in the skin, gastrointestinal tract and lungs, we used intravenous (IV) administration of AgNPs and ionic silver to evaluate their potential systemic toxicity. The study design included a thorough characterization of AgNPs suspensions before use, assessment of tissue distribution by measuring silver concentrations in blood and main organs, and histopathological examination to evaluate the presence of adverse effects and silver localization.

## Results and discussion

### Physicochemical characterization of silver nanoparticles

Commercial AgNPs with a nominal size of 10, 40 and 100 nm were tested in this study. Citrate (CT)- and polyvinylpyrrolidone (PVP)-coated AgNPs were investigated to probe the effect of surface stabilizing agents. Details provided by the manufacturer on the physico-chemical properties of the studied AgNPs are reported in Table 1. The particles were thoroughly characterized before the investigation of their toxicological effects *in vivo* and their accordance to manufacturer’s specifications assessed. A rigorous characterization of the test dispersions is prerequisite to produce data that can help provide scientific answers to regulatory issues, which are impelling for a widely used nanomaterial type such as AgNPs.

**Table 1:** Main physicochemical properties of tested AgNPs provided by the manufacturer and reported in the datasheet

		DLS	UV–Vis		TEM					
Biopure^TM^ Silver nanoparticle	Lot N°	Mean hydrodynamic diameter	λmax	Hmax	Diameter	Variation coefficient	Mass concentration	Particle concentration	Solvent	pH of solution
		(nm)	(nm)	(a.u.)	(mean ± SD) (nm)	(%)	(mg/ml)	(n° of particles/ml)		
10 nm AgNP-CT	DAG1542	na	388	164.9	8.8 ± 1.7	19.6	1.03	3.5*10^14^	2.0 mM citrate	7.3
10 nm AgNP-PVP	DAG1823A	21.3	389	160.2	9.5 ± 1.9	7.5	1.1	2.2*10^14^	MilliQ water	6.9
40 nm AgNP-CT	DAG1176	53.7	411	151.1	40.6 ± 3.0	7.0	1.12	2.7*10^12^	2.0 mM citrate	7.6
40 nm AgNP-PVP	DAG1391	49.3	411	148.2	40.7 ± 4.1	20.2	1.1	2.7*10^12^	MilliQ water	6.7
100 nm AgNP-CT	DAG1186	99.8	495	49.2	99.4 ± 7.0	10.0	1.0	1.9*10^11^	2.0 mM citrate	7.3
100 nm AgNP-PVP	DAG1189	117.0	492	48.2	99.0 ± 5.7	5.8	1.07	1.9*10^11^	MilliQ water	5.9

*CT* sodium citrate, *PVP* polyvinylpyrrolidone, *na* not available (not reported in the datasheet)

Three different techniques were employed, namely Dynamic Light Scattering (DLS), UV-visible (UV–Vis) spectroscopy, and Transmission Electron Microscopy (TEM). First, the hydrodynamic diameter of the particles and their possible aggregation when suspended in the testing medium were evaluated by DLS. The results are summarized in Table 2. Monomodal distributions were observed for 40 nm and 100 nm AgNPs, coated with both CT and PVP. The 10 nm AgNP-CT and 10 nm AgNP-PVP suspensions showed multimodal distributions. In particular, the peaks at 18.1 and 19.6 nm were indicative of isolated nanoparticles in 10 nm AgNP-CT and 10 nm AgNP-PVP, respectively, while larger peaks in both samples suggested the possible presence of aggregates with variable dimensions. However, these large peaks were still detected by DLS even after filtration (0.22 μm pore size), thus indicating their dynamic nature.

**Table 2:** Physicochemical characterization of tested AgNPs. The main findings in AgNPs characterization are reported for each tested sample. For DLS analyses, the mean size of AgNPs is expressed in terms of hydrodynamic diameter, however this parameter is fully informative only for samples with monomodal distributions. Accordingly, the maximum intensity peaks were also reported to describe more comprehensively samples having multimodal distributions (i.e. 10 nm AgNP-CT and 10 nm AgNP-PVP). The pdI provides a measure of particles uniformity. For UV–Vis analyses, the maximum wavelength (λ_max_, *i.e.* the wavelength corresponding to the highest absorbance of AgNPs) and the maximum absorbance value (H_max_) are reported. The λ_max_ and H_max_ values were expressed in nanometer (nm) and arbitrary units (a.u.), respectively. Finally, AgNPs size distributions expressed as Feret diameter (mean ± SD, nm) and variation coefficient (%) were obtained from TEM analysis

Biopure^TM^ Silver nanoparticle	Lot N°	DLS			UV–Vis		TEM	
		Mean hydrodynamic diameter	Max intensity peaks	pdI	λmax	Hmax	Diameter	Variation coefficient
		(nm)	(nm)		(nm)	(a.u.)	(mean ± SD, nm)	(%)
10 nm AgNP-CT	DAG1542	np	18.1– 4046	0.258	392	163.5	8.4 ± 1.5	25.4
10 nm AgNP-PVP	DAG1823A	np	19.6– 111– 4292	0.343	389	163.6	10.8 ± 2.6	24.0
40 nm AgNP-CT	DAG1176	40.1	49.8	0.213	412	152.6	39.3 ± 4.8	12.3
40 nm AgNP-PVP	DAG1391	51.8	67.6	0.251	410	145.7	40.3 ± 5.6	13.9
100 nm AgNP-CT	DAG1186	87.6	102.9	0.148	490	45.1	107.7 ± 10.5	9.8
100 nm AgNP-PVP	DAG1189	104.1	119.4	0.124	491	46.8	105.5 ± 10.9	10.4

*Np* not provided for samples with multimodal distributions

**Fig. 1:**
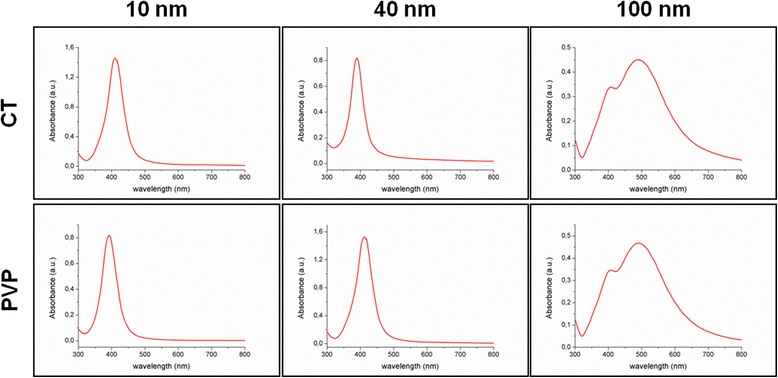
Particle characterization by UV–Vis spectroscopy: full absorbance spectra of the tested silver nanoparticles. The optical density in the 600–800 nm range, which is typical for aggregate absorption, was not detected in any of tested AgNPs, indicating the absence of stable aggregates in these samples

To further investigate the intrinsic features of the putative aggregates detected in 10 nm AgNPs, UV–Vis spectroscopy measurements were performed. AgNPs exhibit a characteristic absorbance maximum in the visible range due to the surface plasmon resonance (SPR) effect [37]. Notably, optical properties of AgNPs are closely related to their morphology, therefore UV–Vis spectroscopy is able to detect any change in size/shape as well as the presence of aggregates. The UV–Vis results are shown in Table 2. The correspondence between the optical properties given by the manufacturer and those measured in our laboratory appeared satisfactory; in particular, no decrease in the maximum absorbance value (H_max_) was observed, indicating absence of aggregates. Then, full absorbance spectra of all samples were considered (Fig. 1). The optical density in the 600–800 nm range, which is typical for aggregate absorption, was not detected in 10 nm AgNP-CT and 10 nm AgNP-PVP, further demonstrating that the presence of stable aggregates in these samples could be excluded [38].

**Fig. 2:**
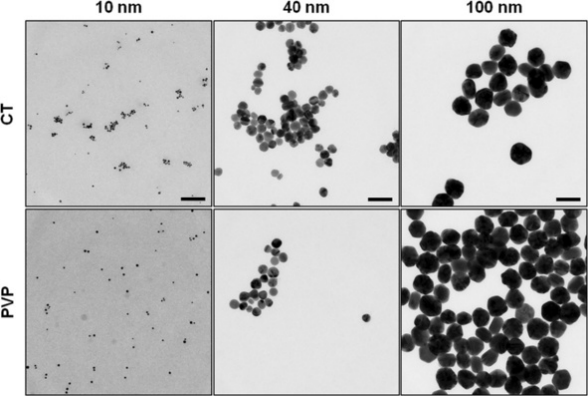
TEM of tested silver nanoparticles. Representative transmission electron micrographs of 10, 40, and 100 nm AgNPs, CT- and PVP-coated. All purchased particles were spherical in shape, and no stable aggregates were visible (scale bar = 100 nm)

Eventually, TEM analysis was performed to assess the shape and primary size distribution of tested AgNPs. All the tested AgNPs were spherical in shape (Fig. 2), and their Feret diameter distributions were in good accordance with data reported by the manufacturer (*p* > 0.05 in all cases) (Table 2).

According to these analyses, both the 10 nm AgNP-CT and the 10 nm AgNP-PVP gave questionable DLS results, since peaks by far larger than 10 nm were detected. Similar findings were recently reported for AgNPs with size lower than 20 nm and 15 nm [21]. Here, the absorbance spectra clearly confirmed the absence of any stable aggregate, since neither the H_max_ decrease nor the absorbance in the 600–800 nm range were visible. TEM analysis further confirmed the absence of aggregates. Therefore, the large peaks detected in the DLS analyses of 10 nm AgNPs were ascribed to dynamic aggregates, which are unstable and do not represent a problem for *in vivo* experiments. In conclusion, the characterization by DLS, UV–Vis spectroscopy, and TEM of stock AgNPs suspensions confirmed the particle size certified by the manufacturer and their suitability for *in vivo* administration.

### Dissolution of silver nanoparticles in mouse serum

The presence of ionic silver in the stock suspensions of CT-coated AgNPs as well as AgNPs dissolution upon interaction with mouse serum was investigated by filtration on membranes with a pore size allowing discrimination of ionic silver from AgNPs. The percentage of ionic silver in the original stock suspensions was found to be negligible (0.002 %, 0.001 %, 0.001 % of total silver for 10, 40 and 100 nm AgNPs, respectively). Time-dependent dissolution of AgNPs in simulated biological conditions is shown in Table 3. As expected, dissolution was greater for smaller particles and increased with time; however even for 10 nm-sized particles at 24 h, the percentage of ionic silver was found to be exceedingly low (0.005 %).

**Table 3:** Time dependent dissolution of AgNPs in simulated biological conditions. Results for ionic silver are expressed as percentage of total silver measured in the stock suspensions

Biopure^TM^ Silver nanoparticle	5 min	10 min	60 min	24 h
10 nm AgNP-CT	0.377*10^−3^	0.510*10^−3^	0.937*10^−3^	5.010*10^−3^
40 nm AgNP-CT	0.049*10^−3^	0.075*10^−3^	0.176*10^−3^	1.235*10^−3^
100 nm AgNP-CT	0.005*10^−3^	0.016*10^−3^	0.038*10^−3^	0.366*10^−3^

The low dissolution of AgNPs when incubated in mouse serum is in accordance with other studies, which put forward that following introduction into a physiological environment the ability of AgNPs to release Ag^+^ is likely inhibited due to the formation of the protein corona [39]. Interaction of protein thiol groups with the charged surface of AgNPs in a medium with a high ionic strength is at the basis of the sulfidation process that leads to an extensive decrease of the dissolution rate *in vitro*, causing also the formation of nanobridges between particles, and has the potential to stabilize them *in vivo* [40]. Formation of the protein corona decreases the extracellular dissolution of AgNPs leading to cellular uptake of particles, which may lose the corona after internalization resulting in an exacerbated release of Ag ions and toxicity [39].

Dissolved silver ions are likely to immediately react with chlorine in serum to form AgCl [41]. On the other hand, it cannot be ruled out that a minor part of the released silver ions might have been complexed with high affinity S-containing proteins and thus been excluded by the membrane used, leading to a possible underestimation of the level of released Ag^+^ [42]. However, even though the method used for studying particle dissolution may have not provided an entirely accurate measure of ionic silver formation in physiological conditions – all available methods have some limitations in this respect [43] – it clearly shows that extracellular dissolution of the AgNPs used in this study is very limited and animals dosed with AgNPs were internally exposed to particulate and not ionic silver.

### *In vivo* study

Mice were intravenously (IV) injected with CT- or PVP-coated AgNPs of different sizes at a single dose of 10 mg/kg body weight (bw). For comparison, a group was treated with silver acetate (AgAc), used as source of silver ions, at a dose of 15.5 mg/kg bw, containing the equivalent dose of 10 mg Ag/kg bw. The body weight of each mouse was measured before treatment and at sacrifice. Mice were euthanized 24 h after the treatment, and blood and organs were collected for silver quantification and histological examination.

In this study, the IV route of administration was selected in order to avoid the variability in absorption from the different exposure sites and identify the potential target organs for distribution and toxicity of particles of different size and coating as well as of ionic silver. Although IV administration is not considered a relevant route of exposure to AgNPs for the consumer, this route of exposure can provide valuable information about the *in vivo* behavior of AgNPs crossing the primary barriers (skin, lung, gastrointestinal tract) and entering blood circulation, or administered for clinical purposes (e.g. intravascular medical devices, wound dressings, imaging, drug delivery) [44–47]. In many studies silver was detected in the main organs after exposure to AgNPs by different routes [15, 16, 21, 25, 48, 49], but only few studies investigated the presence of silver particles in tissues after oral exposure in rats [21, 48]. Loeschner et al. [48] found silver granules by using TEM in the same size range of administered AgNPs in the intestine (basal lamina, macrophages, connective tissue of submucosa), but not in the liver, after oral administration of 14 nm AgNPs and AgAc (9 mg/kg b.w. of silver. Another study [21], by using single particle ICP-MS, revealed the presence of NPs in the examined organs (gastrointestinal tract, liver, spleen, lungs) after oral administration of 15–20 nm AgNPs (90 mg/kg b.w) and AgNO_3_ (9 mg/kg). In both studies, nanoparticles were found also in the group treated with Ag^+^, indicating that nanoparticles can be formed *in vivo* from soluble silver. Even though there is still little information on the ability of AgNPs to be absorbed, and distribute systemically after dermal application, inhalation, or ingestion, a definite conclusion about the complete dissolution of AgNPs before reaching the blood circulation or within the blood cannot be drawn as well, leaving open the question about the potential effects of AgNPs in case they enter (even partially) blood circulation.

A dose of 10 mg/kg bw was chosen since it was in the range of doses used in previous IV studies without eliciting relevant adverse effects in animals [22, 23, 30, 31, 50]. A dose of 10 mg/kg bw in mice is equivalent to a human dose of 0.81 mg/kg bw, corresponding to approximately 50 mg for a human of 60 kg, according to guidelines for dose translation from animals to humans [51].

### Animal behavior, body and organ weights

Immediately after administration of AgNPs and AgAc and during the following hours, all mice appeared healthy and no abnormal behavior was observed. However, 24 h after the treatment two mice (one mouse treated with 10 nm AgNP-CT and another one treated with 10 nm AgNP-PVP) were found dead. Complications related to the injection procedure or formation of large aggregates after administration were reasonably ruled out given the delayed onset of mortality and results of later histopathological evaluation (i.e. absence of thromboembolic lesions associated with silver aggregates). At sacrifice, no significant differences in body weight gain and organ weights were recorded between CT- and PVP-coated AgNPs of the same size (Additional file 1: Table S1). In mice treated with 10 nm AgNPs a significant difference in percentage of weight loss and relative spleen weight were observed compared to control and 40 and 100 nm AgNP-treated mice (Table 4). No other significant differences were observed in relative organ weights between treated and control mice.

**Table 4:** Body weight gain and relative organ weight (%) after IV administration of 10 mg silver/kg. Data are expressed as means ± SD

Group	n	Body weight gain	Spleen	Liver	Lung	Kidney	Brain
Control	3	−3.3 ± 5	0.45 ± 0.1	6.61 ± 0.8	0.66 ± 0.0	1.80 ± 0.2	1.77 ± 0.1
10 nm AgNP	6	−12.1 ± 4.1*	0.67 ± 0.0*	7.23 ± 1.1	0.76 ± 0.1	1.75 ± 0.1	1.81 ± 0.2
40 nm AgNP	6	−1.3 ± 2.8^++^	0.54 ± 0.1^++^	6.87 ± 0.6	0.65 ± 0.1	1.79 ± 0.3	1.78 ± 0.1
100 nm AgNP	6	−0.1 ± 5.0^++^	0.40 ± 0.1^++^	6.97 ± 0.2	0.63 ± 0.1	1.80 ± 0.1	1.65 ± 0.1
AgAc	3	−6.3 ± 3.3	0.47 ± 0.0^+^	6.99 ± 0.0	0.80 ± 0.1	1.92 ± 0.2	1.78 ± 0.2

*p <0.05 vs Control; + *p* < 0.05 ++*p* < 0.01 vs 10 nm AgNP

### Tissue distribution and localization of silver

Distribution and localization of silver in the different organs 24 h after IV exposure to CT- and PVP-coated AgNPs of three different sizes, and AgAc, were evaluated using two distinct but complementary approaches, i.e. inductively coupled plasma mass spectrometer (ICP-MS) and autometallography (AMG) staining. ICP-MS was used to quantitatively measure the total silver concentration in spleen, liver, lung, kidney, brain, and blood, while AMG was used to qualitatively assess silver localization within the sampled organs.

In the control group, silver was present at background levels as shown by ICP-MS data for the examined organs (Additional file 1: Table S2). In the treated animals, at 24 h after administration the silver concentration in blood was drastically reduced in all groups compared to the peak concentrations expected on the basis of the administered dose, in agreement with previous studies of kinetics performed after one single IV administration of 120 mg/kg of 15 nm AgNPs [22] and 0.8 mg/kg of 20, 80, and 110 nm AgNPs [30]. In the blood of 10 nm AgNPs-treated mice silver was approximately 1.5 times and 3.5 times higher than in mice treated with 40 nm and 100 nm AgNPs, respectively (Additional file 1: Table S2). Since previous studies demonstrated a rapide decline (minutes to few hours) of silver blood levels after IV injection of AgNPs regardless of nanoparticle size [22, 30, 50], the greater silver concentration of 10 nm AgNPs-treated mice 24 h after exposure might indicate an increased and earlier redistribution of silver from organs to blood in the case of 10 nm AgNPs compared to larger ones. The results obtained for 10 nm AgNPs are in agreement with those of a IV study on CT-coated AgNPs having a similar size (8 nm), administered to rats at the same dose of the present study (10 mg/kg bw); after an initial decrease (up to 4 h post injection) blood silver levels increased again and did not decrease during the experimental period until 96 h [50].

**Fig. 3:**
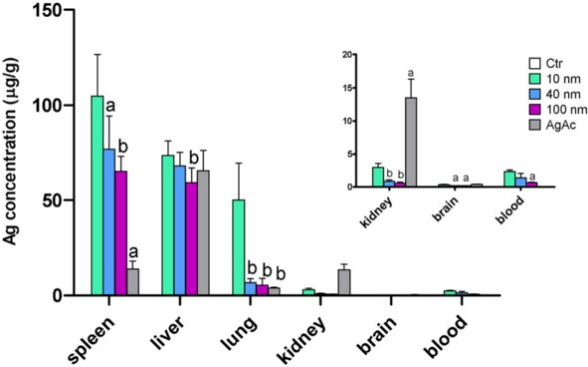
Silver tissue concentration after IV administration of AgNPs and AgAc in mice. Data are expressed as means ± SD. Statistical significance: a = *p* < 0.05; b = *p* < 0.01 vs 10 nm

For all particle sizes, regardless of their coating, the highest silver concentrations were found in the spleen and liver, followed by lung, kidney and brain (Fig. 3, and Additional file 1: Figure S1). These results are in line with previous studies that investigated tissue distribution of AgNPs 24 h after IV administration [22, 30, 50]. When considering the percentage of the administered silver dose recovered after administration, for all tested AgNPs approximately 40 % of the administered dose was found in the liver, which resulted to be the main target organ of silver distribution, followed by spleen, lung, kidney, and brain (Table 5). Most of the silver that reaches the blood is filtered by the liver and excreted into the bile [50], while the remaining circulating particles distributed to organs containing large numbers of phagocytic cells, such as liver, spleen, and lung, which are devoted to clearing foreign body particles from the circulating blood. The role of macrophages in general, and Kupffer cells in particular, in clearance and accumulation of nanoparticles was the same observed after intravenous administration of other metallic nanoparticles (e.g. gold NPs) [52, 53].

**Table 5:** Percentage of recovered silver dose in mice after IV administration of 10 mg silver/kg. Data are expressed as means ± SD

Group	n	Spleen	Liver	Lung	Kidney	Brain
10 nm AgNP-CT	3	6.6 ± 1.2	52.0 ± 4.7	4.2 ± 1.1	0.5 ± 0.1	0.056 ± 0.015
10 nm AgNP-PVP	3	5.3 ± 1.0	41.1 ± 2.3	1.5 ± 1.2	0.4 ± 0.1	0.049 ± 0.016
40 nm AgNP-CT	3	3.6 ± 1.1	46.1 ± 2.5	0.5 ± 0.1	0.1 ± 0.1	0.037 ± 0.007
40 nm AgNP-PVP	3	3.1 ± 1.1	45.5 ± 1.9	0.3 ± 0.1	0.2 ± 0.0	0.035 ± 0.009
100 nm AgNP-CT	3	2.7 ± 0.4	36.7 ± 0,3	0.5 ± 0.1	0.1 ± 0.0	0.029 ± 0.004
100 nm AgNP-PVP	3	2.5 ± 0.6	45.7 ± 1.1	0.2 ± 0.0	0.1 ± 0.0	0.028 ± 0.003
AgAc	3	0.6 ± 0.2	42.6 ± 4.5	0.3 ± 0.1	2.4 ± 0.6	0.064 ± 0.011

With exception of the liver, where similar silver concentrations were identified regardless of the source of administered silver (AgNP or silver ions), in the other examined organs silver concentrations were higher in mice treated with 10 nm AgNPs (including both CT- and PVP-coated particles) than in mice treated with larger particles (40 nm, 100 nm), and concentrations decreased with increasing size of AgNPs. Since the silver concentrations found in the brain and kidney were comparable or lower than those found in the blood, silver contribution of residual blood contained in these organs cannot be completely ruled out.

As regards the role of coating on silver tissue distribution, no significant differences were found between CT- and PVP-coated AgNPs of the same size, in line with previous studies [21]. However, Principal Component Analysis (PCA) highlighted a trend of CT-coated 10 nm AgNPs to cluster separately from PVP-coated 10 nm AgNPs (right side of the first Principal Component, PC1) likely due to the higher silver concentrations of the 10 nm AgNP-CT in spleen, lungs, liver, and, to a lesser extent, in kidneys compared to 10 nm AgNP-PVP (Additional file 1: Figure S2). PCA thus revealed that the coating might have a potential effect of on tissue distribution at least for the smallest (10 nm) AgNPs.

Silver localization within organs was evaluated histologically. In H&E stained sections intracytoplasmic black granular pigment was multifocally (and often barely) visible in the liver (along the sinusoids), spleen (marginal zone and red pulp), and lungs (alveolar septa) of all AgNP-treated mice, but not in AgAc-treated mice (Additional file 1: Figure S3).

**Fig. 4:**
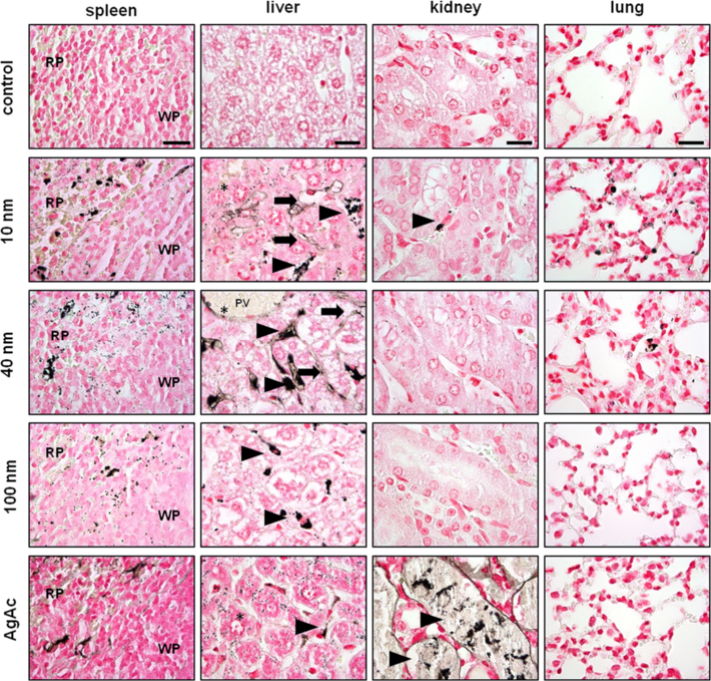
Histological evaluation of silver tissue localization by AMG. Representative images of spleen, liver, kidney, and lung (scale bar = 20 μm), from vehicle- (control), AgNP (10 nm, 40 nm, 100 nm), and AgAc-treated mice. In the spleen, silver was localized within the cytoplasm of macrophages in the marginal zone of the white pulp (WP) and in the red pulp (RP). In the liver, the cellular localization of silver varied depending on the size of the AgNPs. In 10 nm AgNP-treated mice, silver was present in the cytoplasm of Kupffer cells (arrowhead), sinusoidal endothelial cells (arrow) and hepatocytes (*). In 40 nm AgNPs-treated mice, silver was localized in the cytoplasm of portal endothelial cells (*), sinusoidal endothelial cells (arrow) and Kupffer cells (arrowhead). In 100 nm AgNPs-treated mice, most of silver was concentrated in the cytoplasm of Kupffer cells (arrowhead). In AgAc-treated mice, silver was present in the cytoplasm of hepatocytes (*), and Kupffer cells (arrowhead). In the kidney, occasional silver-containing cells were observed in the renal interstitium of 10 nm AgNP treated-mice (arrowhead), and large amounts of silver were identified in necrotic tubules of AgAc-treated mice (arrowhead). In the lung, scattered silver-containing cells were found in the alveolar septa of 10 nm and 40 nm AgNP-treated mice

The tissue localization of silver was better detailed after AMG staining (Fig. 4), which enhances the silver present within the tissues, providing a rapid, cost-effective histochemical means of detecting the distribution of silver within organs [54]. In AgNP-treated mice, regardless of the particle size and coating, the organs showing the greatest silver accumulation were the spleen and liver in agreement with the results of total silver quantification obtained by ICP-MS. Occasionally, enhanced silver clusters were found in the lung and occasionally in the kidney (mainly in 10 nm AgNPs-treated mice), and none in the brain. In the spleen, silver was localized within the cytoplasm of macrophages in the marginal zone of the white pulp and in the red pulp, and occasionally within splenic endothelial cells. In the liver most of the silver was found in the cytoplasm of Kupffer cells along the sinusoids, and occasionally within sinusoidal and portal endothelial cells, and more rarely within hepatocytes, in accordance with previous studies [23, 31, 55]. A sort of size-dependent pattern of silver distribution in the liver was observed, suggesting that silver uptake by Kupffer cells is almost exclusive after administration of 100 nm AgNPs, while administration of 10 nm and 40 nm AgNPs resulted in silver uptake also by endothelial cells, and hepatocytes, in addition to Kupffer cells. Only occasionally, the silver was identified in the gall bladder of 10 nm AgNP-treated mice (within gall bladder epithelial cells and endothelial cells of blood vessels). In the lung, scattered silver containing cells were found in the alveolar septa, either within the capillaries or the interstitium. In the kidney, only in mice treated with 10 nm AgNPs there were occasional silver-containing cells in the glomerular tufts, and renal interstitium, morphologically consistent with circulating leukocytes (monocytes) that were also occasionally observed in the other examined organs. No enhanced silver clusters were identified in the brain sections of treated mice. This lack of histological identification of silver in the brain is consistent with the very small concentrations of silver detected in this organ by ICP-MS.

In mice treated with AgAc silver concentrations determined by ICP-MS were similar in the liver and brain, but significantly lower in the spleen and lung, and higher in the kidney compared to mice treated with 10 nm AgNPs (Fig. 3). Differently from AgNP-treated mice, no black granular deposits (reminiscent of silver) were found throughout the examined tissues stained with H&E. In AMG stained sections, silver enhanced clusters were identified in the liver (within Kupffer cells and hepatocytes), spleen (marginal zone and red pulp), and kidney (at level of degenerated/necrotic tubules) (Fig. 4). No silver was identified in the lung and brain. Organs from AgAc-treated mice showed higher values than those from the control and AgNP-treated mice on PC2, due to the higher silver concentration in the kidney (Additional file 1: Figure S2). Overall these results suggest that silver ions have a different biodistribution pattern, and likely also of cell uptake, and excretion (i.e. enhanced role of renal excretion in addition to biliary excretion) compared to nanoparticles. This difference in kinetics was reflected also by the identification of distinct target organ of toxicity for silver ions and AgNPs, as discussed below.

**Fig. 5:**
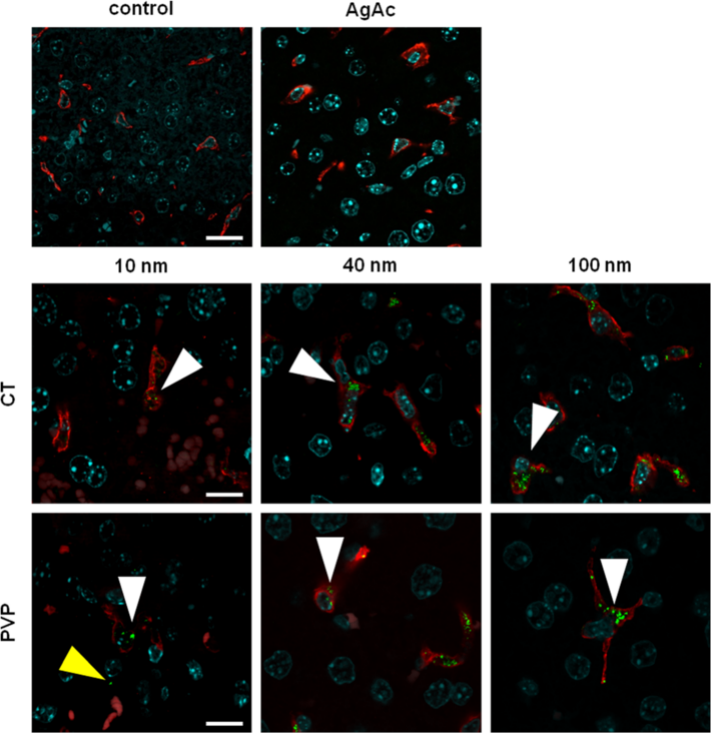
Localization of silver in the liver evaluated by confocal microscopy. Representative images of liver (scale bar = 20 μm), from vehicle- (control), AgAc, and CT- and PVP-coated AgNP (10 nm, 40 nm, 100 nm)-treated mice. Aggregates of silver (green) are found within the cytoplasm of Iba-1+ Kupffer cells (red) (white arrows) only in AgNP-treated mice. Rarely, small numbers of silver aggregates are found outside Kupffer cells (yellow arrow), most likely in the cytoplasm of endothelial cells or hepatocytes. Nuclei are counterstained with DAPI (cyan)

Immunostaining of sections with IBA1 (a pan-macrophage marker) further confirmed that most of silver-containing aggregates were present within the cytoplasm of Kupffer cells in the liver, in all AgNP-treated groups (Fig. 5). The amount and size of silver-containing aggregates within macrophages decreased with decreasing size of AgNPs. In 10 nm AgNPs-treated mice only scattered and very small aggregates were found throughout the liver, while no visible silver-containing aggregates were found in the liver of AgAc treated mice, despite the identification of silver by AMG staining within Kupffer cells and hepatocytes. This result may suggest a different intracellular behavior of silver depending on its form and size.

Since ICP-MS does not provide information about the soluble or particulate form of silver detected within the tissue, it is not possible to know whether the silver-containing aggregates identified histologically in this study were composed of silver nanoparticles or silver salts formed upon interaction of silver ions with sulphur and selenium, as previously described in the intestine of rats following oral administration of AgNPs and AgAc [48], or in the skin of patients with argyria [56, 57]. Additional analyses, e.g. employing TEM with energy dispersive x-ray spectroscopy (EDX), and Single Particle ICP-MS (not for the 10 nm AgNPs, which are below the size detection limit of the technique) should be taken into consideration in future studies, to assess the composition and subcellular localization of silver within tissues, allowing to better understand the intracellular fate of AgNPs and/or silver ions.

### Toxicity

**Fig. 6:**
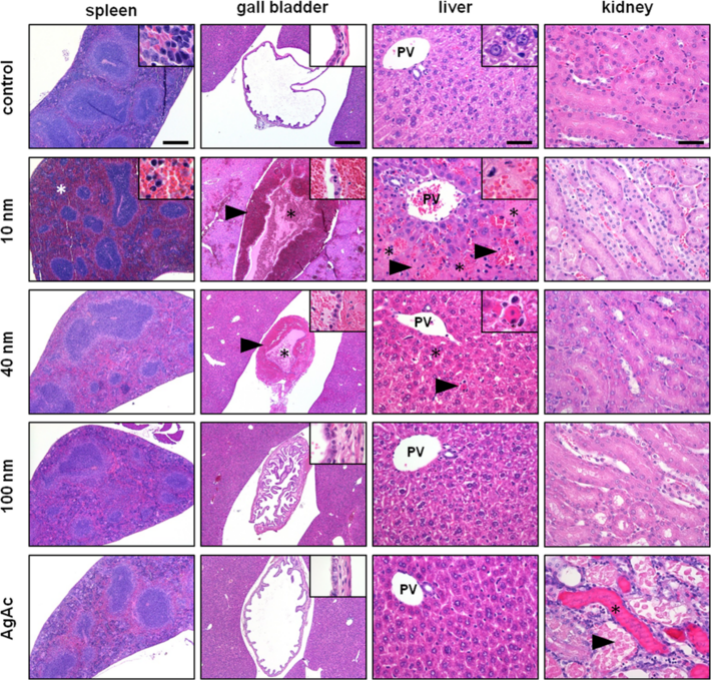
Histological evaluation of adverse effects after single IV administration of AgNPs and AgAc in mice. Representative images of spleen (scale bar = 500 μm), gall bladder (scale bar = 500 μm), liver (scale bar = 50 μm), and kidney (scale bar = 50 μm) from vehicle- (control), AgNP (10 nm, 40 nm, 100 nm), and AgAc-treated mice. No relevant changes were present in the examined organs of control mice. In the spleen, a severe hyperemia of the red pulp was present in 10 nm-treated mice (*). In the gall bladder, severe intraluminal (*) and mural (arrowhead) hemorrhages were found in 10 nm AgNPs-treated mice, and in a single mouse treated with 40 nm AgNPs, while in 100 nm-treated mice only mild mural edema and hyperemia were occasionally evident. In the liver, in 10 nm AgNP-treated mice marked midzonal hepatocellular necrosis (*) and hemorrhages (arrowhead) were present, while in 40 nm-treated mice only occasional single cell necrosis (arrowhead, and inset) and early signs of coagulative periportal necrosis (*) were found (PV = portal vein). In the kidney, in AgAc-treated mice marked tubular necrosis (arrowhead) with intraluminal hyaline casts (*) were present

Two mice out of six were found dead 24 h after treatment with 10 nm AgNPs. No relevant gross changes were observed in the examined mice, except for a moderate splenomegaly in mice treated with 10 nm AgNPs. Histological AgNP-related lesions were observed in the spleen, liver, and gall bladder of AgNP-treated mice (Fig. 6). No relevant differences were found between CT- and PVP-coated particles of the same size. Overall the prevalence and severity of lesions (in particular those involving liver and gall bladder) were size-dependent, with mice treated with 10 nm AgNPs most frequently and severely affected compared to 40 nm and 100 nm AgNP-treated mice that had milder or negligible lesions, respectively (Tables 6 and 7). Splenic hyperemia was present in all mice treated with AgNPs, but only in mice treated with 10 nm-sized particles it was particularly pronounced, and thus likely responsible for inducing splenomegaly, as well as the increased relative spleen weight observed in this group. Mice treated with 10 nm AgNPs were affected by diffuse and severe midzonal hepatocellular necrosis and hemorrhage, multifocal peribiliary microhemorrhages, occasional portal vein endothelial damage (i.e. endothelial sloughing, subendothelial hemorrhages, intraluminal fibrin thrombi) (Additional file 1: Figure S4), and diffuse mural and intraluminal hemorrhage of the gall bladder. Among mice treated with 40 nm AgNPs only one out of six had early periportal coagulative necrosis, scattered hepatic single cell necrosis, and gall bladder severe mural and intraluminal gall bladder hemorrhage. In the other mice treated with 40 nm AgNPs, the hepatic lesions were not observed and gall bladder lesions were milder, usually consistent with mural hyperemia and/or edema. Mice treated with 100 nm AgNPs had only occasional and mild gall bladder mural hyperemia and/or edema. No relevant pathological changes were observed in the lung, kidney and brain of AgNP-treated mice, and in all the examined organs of control mice.

**Table 6:** Prevalence of histopathological lesions in mice following IV exposure to 10 mg silver/kg. Data are expressed as number of mice with lesions/total number of examined mice per group (%)

Group	Spleen	Liver	Gall bladder	Lung	Kidney	Brain
Control	1/3 (33 %)	0/3 (0 %)	0/3 (0 %)	0/3 (0 %)	0/3 (0 %)	0/3 (0 %)
10 nm AgNP-CT	3/3 (100 %)	3/3 (100 %)	3/3 (100 %)	0/3 (0 %)	0/3 (0 %)	0/3 (0 %)
10 nm AgNP-PVP	3/3 (100 %)	3/3 (100 %)	3/3 (100 %)	0/3 (0 %)	0/3 (0 %)	0/3 (0 %)
40 nm AgNP-CT	3/3 (100 %)	1/3 (33 %)	2/3 (67 %)	0/3 (0 %)	0/3 (0 %)	0/3 (0 %)
40 nm AgNP-PVP	2/3 (67 %)	0/3 (0 %)	1/3 (33 %)	0/3 (0 %)	0/3 (0 %)	0/3 (0 %)
100 nm AgNP-CT	1/3 (33 %)	0/3 (0 %)	2/3 (67 %)	0/3 (0 %)	0/3 (0 %)	0/3 (0 %)
100 nm AgNP-PVP	1/3 (33 %)	0/3 (0 %)	0/3 (0 %)	0/3 (0 %)	0/3 (0 %)	0/3 (0 %)
AgAc	0/3 (0 %)	0/3 (0 %)	0/3 (0 %)	0/3 (0 %)	3/3 (100 %)	0/3 (0 %)

**Table 7:** Grading of most relevant histopathological lesions in mice after IV administration of 10 mg silver/kg. Results are expressed as median score per group (range) (*n* = 3)

Group	Hepatobiliary tract				Spleen	Kidney
	Hepatocellular necrosis and hemorrhage	Peribiliary hemorrhage	Portal vein endothelial damage	Gall bladder hemorrhage	Red pulp hyperemia	Tubular necrosis
Control	0 (0–0)	0 (0–0)	0 (0–0)	0 (0–0)	1 (0–2)	0 (0–0)
10 nm AgNP-CT	4 (3–4)	2 (1–4)	1 (0–4)	2 (2–4)	2 (2–3)	0 (0–0)
10 nm AgNP-PVP	4 (2–4)	2 (1–4)	2 (0–4)	2 (1–4)	3 (3–4)	0 (0–0)
40 nm AgNP-CT	0 (0–1)	0 (0–1)	0 (0–0)	1 (1–4)	2 (2–3)	0 (0–0)
40 nm AgNP-PVP	0 (0–0)	0 (0–0)	0 (0–0)	0 (0–1)	2 (1–2)	0 (0–0)
100 nm AgNP-CT	0 (0–0)	0 (0–0)	0 (0–0)	1 (0–1)	0 (0–1)	0 (0–0)
100 nm AgNP-PVP	0 (0–0)	0 (0–0)	0 (0–0)	0 (0–0)	1 (0–2)	0 (0–0)
AgAc	0 (0–0)	0 (0–0)	0 (0–0)	0 (0–0)	0 (0–0)	4 (3–4)

A completely different pathological scenario was observed after administration of AgAc. Hepatobiliary lesions were not observed, whereas a marked acute renal tubular necrosis and apoptosis with intraluminal accumulation of hyaline casts was found in all treated mice. Presence of renal lesions, associated with identification of silver in the affected renal tubules and relevant silver concentrations in the kidney of AgAc-treated mice indicates that dissolved silver ions resulted in an increased renal silver distribution compared to AgNPs, and that renal lesions were likely secondary to the renal excretion of silver.

Although liver is considered one of the most important target organs for AgNPs, given its capacity of AgNP accumulation and its role in biliary excretion of AgNPs [49], so far hepatotoxic effects of AgNPs have been inconstantly demonstrated in *in vivo* studies, either after IV, oral, or inhalation exposure to AgNPs. In particular, hepatotoxicity was generally recognized through changes in the blood biochemical parameters of liver function (such as ALT, AST, ALP, and cholesterol) [16, 17, 23, 58], while only occasionally mild histopathological changes were reported, such as biliary hyperplasia, fibrosis, hepatocellular vacuolar degeneration, and rarely necrosis [15, 16, 22]. The lack of overt hepatic histopathological lesions in previous studies that used small-sized particles could be related to a poor dispersion of AgNPs in the administered suspensions [22], since agglomeration of particles can affect bioavailability by reducing the rate of degradation or cell uptake [8]. According to our characterization of AgNPs, we administered well-dispersed 10 nm-sized particles. Another aspect is that most of earlier *in vivo* studies of distribution and toxicity of AgNPs have been performed in rats and not in mice. Further investigation should be performed in order to assess whether there is a different sensitivity between these two species.

In addition to the severity of the hepatic lesions found in mice treated with 10 nm AgNPs, also the pattern of hepatocellular necrosis observed is very unusual. Midzonal necrosis is the least common histologic pattern of hepatocellular necrosis following exposure to hepatotoxicants [59]. Because hepatocytes are extremely variable in their metabolic capacity and oxygen tension, midzonal necrosis of hepatocytes is presumably dictated by an unique susceptibility of these hepatocytes based on their location within the hepatic lobule [60]. The hemorrhage observed in the gall bladder of mice treated with 10 nm AgNPs and occasionally in mice treated with 40 nm AgNPs is another very unusual finding. We can speculate that gall bladder mural hemorrhage, as well as portal peribiliary hemorrhages, could be the result of endothelial damage secondary to a massive elimination of AgNPs in the bile, which is considered the main route of silver excretion following IV administration of AgNPs [31, 50]. The hypothesis of particle-induced endothelial damage is further corroborated by the presence of endothelial damage found in the intrahepatic branches of portal veins that may have occurred after intestinal re-absorption of AgNPs excreted in the bile. Another concurrent factor in the pathogenesis of gall bladder hemorrhage could be the anti-platelet properties of AgNPs that may have impaired a prompt clotting response [61].

In this study relevant size-dependent adverse effects were observed 24 h after IV administration of AgNPs in mice. First of all, and differently from previous studies where AgNPs were administered IV (up to a dose of 120 mg/kg bw) [22, 23, 30, 31, 50], mortality was observed in 2 out of 6 mice treated with the smallest particles (10 nm AgNPs), regardless of the coating. The most striking finding was the marked hepatotoxicity demonstrated by 10 nm AgNPs (both CT and PVP-coated), associated with a massive hemorrhage of the gall bladder. These lesions (in particular the hemorrhage) were especially severe in the two mice found dead, and can thus be regarded as the most likely cause of death. Of note, administration of AgNPs of different sizes and silver acetate resulted in similar liver content of total silver (as determined by ICP-MS), indicating that hepatobiliary toxicity induced by 10 nm AgNPs was not related to the mass of silver contained in the liver, but rather it was imputable to their small size. Size-dependent toxicity of AgNP was previously demonstrated in *in vitro* [11, 13, 14, 36], as well as in *in vivo* studies using the respiratory route of administration [32, 33]. Our results further support the importance of nanoparticle size for AgNP toxicity *in vivo*. Release of silver ions following oxidation of particle surface after administration of AgNPs is believed to constitute the major mechanism contributing to AgNP toxicity and since dissolution increases with decreasing size of AgNPs [33], this appears to be the determinant of the enhanced toxicity of smaller AgNPs compared to larger ones. The most obvious explanation for increased dissolution of smaller particles, compared to an equivalent mass concentrations of larger particles, is their higher number (in this study, the number of administered 10 nm AgNPs was about 100 fold greater compared to 40 nm AgNPs) and consequent larger surface area per unit mass. The results of our study however demonstrated that systemic availability of silver ions is not responsible for the *in vivo* hepatobiliary toxicity observed in mice treated with 10 nm AgNPs, because dissolution of 10 nm AgNPs in mouse serum was very low (0.005 %) and administration of AgAc resulted in a distinct target organ of toxicity (kidney instead of hepatobiliary system). Cell-type-dependent toxicity of silver ions and AgNPs was previously demonstrated in *in vitro* studies. Furthermore, recent *in vitro* studies demonstrated that toxicity of AgNPs rather than being caused by silver ions liberated in the culture medium is actually dependent on their intracellular release [35, 62, 63]. According to the recently proposed general mechanism for toxicity of metal-containing nanoparticles, the so-called “Lysosome-Enhanced Trojan Horse effect”, toxicity of these particles occurs after cellular uptake by endocytosis and it is mediated by enhanced intracellular release of ions secondary to the acidic corrosion within lysosomes [62]. Increased toxicity of smaller AgNPs is then explained by their increased number and reactive surface area, resulting in increased intracellular release of silver ions. The fact that the entry of AgNPs into cells is size-dependent contribute to further enhance the toxic potential of small particles, owing to their higher bioavailability to cells [11, 13, 14, 35].

In our study, coating of AgNPs with CT and PVP did not have a detectable effect on toxicity indicating that particle size is more important than coating to predict potential adverse effects of silver nanoparticles, at least for the coatings tested herein. Coating-dependent toxic effects were demonstrated *in vitro* [9, 10], and only in the lungs *in vivo* [32, 33]. It can be speculated that in *in vivo* studies, including ours, coating has a minor effect, if any, as a consequence of the formation of a corona after the adsorption of proteins (and other biomolecules) on the surface of particles once they enter in contact with biological fluids [28]. The protein corona might mask the effect of the different particle coatings that is evident in *in vitro* studies. This is in agreement with the recent view that to predict the biological behavior of nanoparticles the fingerprinting of the protein corona is more accurate than the sole characterization of their physicochemical properties, since the protein corona confers to nanoparticles a ‘biological identity’ that is strongly dependent but distinct from its ‘synthetic identity’ [64, 65].

## Conclusions

With this study, the influence of two distinct nanoparticle properties, size and coating, was comprehensively investigated in an *in vivo* model. We observed that tissue distribution and toxic effects of AgNPs in mice after IV administration are strongly size-dependent, while coating (CT or PVP) did not have a sizeable impact on tissue distribution and toxicity. Overall, the smallest (10 nm) particles resulted in higher silver tissue distribution and caused overt acute adverse effects (centered on the hepatobiliary tract) compared to the larger ones (40 and 100 nm). Overall, these results suggest that in the safety assessment of silver particles the effect of size has to be carefully considered, with a focus on small particles (≤10 nm). Comparison with AgAc revealed a distinct pattern of tissue distribution and toxicity between AgNP and silver ions, indicating that the *in vivo* effects of AgNPs are not attributable merely to the *in vivo* release of silver ions in circulating blood but are strictly related to the nanoparticulate form.

## Methods

### Characterization of silver nanoparticles

Suspensions of BioPure™ Silver Nanoparticles (AgNPs) of 10, 40 and 100 nm in size, coated with either citrate (CT) or polyvinylpyrrolidone (PVP), were purchased from NanoComposix (San Diego, USA). All the suspensions were supplied at a concentration of about 1.0 mg/ml. BioPure™ AgNPs were chosen because they were guaranteed to be sterile and with an endotoxin level lower or equal to 2.5 EU/ml. The suspending solvents of CT- and PVP-coated AgNPs were 2.0 mM sodium citrate and Milli-Q water (Millipore), respectively. For particle characterization, the CT and PVP-coated AgNPs were diluted with 2.0 mM sodium citrate (cod. W302600, Sigma-Aldrich) buffer and Milli-Q water, respectively. When necessary, samples were sonicated (Elmasonic S 30 H) for up to 30 s, in accordance with the manufacturer’s instructions. In order to prevent contamination, measurements were run using disposable plastic cuvettes. The AgNPs were tested immediately after their delivery and *in vivo* experiments were run in the following week. In the meanwhile, the AgNPs were stored at +4 °C, according to manufacturer’s instructions.

#### Dynamic Light Scattering (DLS)

The actual size of AgNPs in dispersion was measured by DLS. Measurements were performed with a Malvern Zetasizer Nano ZS90 instrument operating with a light source wavelength of 532 nm and a fixed scattering angle of 90°. All the nanoparticles were diluted 1:100 with the exception of 10 nm-sized AgNPs. Indeed, due to their small size, the 10 nm AgNPs presented increased absorption and lower scattering intensity compared to 40 nm and 100 nm AgNPs. Accordingly, the 10 nm AgNPs were diluted 1:50. All measurements were run at room temperature for at least three times.

#### UV-Visible (UV–Vis) Spectrophotometry

The UV–Vis spectra were acquired in the 300–800 nm range using a DU730 Beckman Coulter Spectrophotometer. All the nanoparticles were diluted 1:100 with the exception of 10 nm AgNPs, which were diluted 1:200 because of their increased UV–Vis absorbance with respect to larger nanoparticles. All measurements were run at room temperature for at least three times.

#### Transmission Electron Microscopy (TEM)

Formvar coated copper TEM grids (cod. PE1GC300, Pelco) were pre-treated with 20 μl of poly-L-lysine 0.01 % (w/v) (Sigma Aldrich) for 15 min. After washing twice with MilliQ water, 3 μl of AgNPs suspensions were deposited onto the grid for 5 min and then rinsed with 3 μl of 2-propanol (Sigma Aldrich). According to the manufacturer’s advice, 100 and 40 nm AgNPs were used at the concentration of 1.0 mg/ml, while 10 nm AgNPs were diluted up to 0.1 mg/ml before use. The grids were allowed to dry overnight at room temperature in a covered crystallizing dish. TEM (FEI Tecnai G2, Eindhoven) images were analyzed with the ImageJ software (http://imagej.nih.gov/ij/) to obtain the nanoparticles dimensional distribution. In particular, small objects due to background and overlapping nanoparticles were omitted by using proper cut-off filters and Feret diameter (intended as the larger diameter of the NP projection) was used to evaluate the size of the particles. For each sample, a minimum of about 250 nanoparticles was considered.

#### Dissolution study

CT-coated particles were selected for this investigation given the higher stability and lower dissolution generally showed by PVP-coated AgNPs [66, 67]. Dissolution of CT-coated AgNPs of 10, 40 and 100 nm was ascertained by ultrafiltration, using a PES spin filter membrane (Vivaspin 500, 3 kDa MWCO, Sartorius, Göttingen, Germany) and centrifugation at 15000 g for 20 min, followed by quantification of silver in the filtrates. The concentration of ionic silver was measured both in the AgNPs stock suspensions and in conditions simulating AgNPs interaction with biological fluids. For the latter purpose, each stock suspension was spiked to mouse serum (Euroclone, Milan, Italy) in order to provide the mass concentration of AgNPs of a single dose of 10 mg/kg bw, which approximately corresponded to a 1:5 dilution (v/v) of the original AgNPs suspension. Spiked samples and serum blank were prepared in triplicate and incubated at 37 °C under agitation for 5, 10, 60 min and 24 h. Ionic silver was assessed for each timepoint. For ICP-MS determination of ionic silver, filtrates (prepared in triplicate) were vigorously shaken before further dilution and analysis as described in the section “Determination of silver”. In addition to ionic silver, each stock suspension of CT-coated AgNPs was characterized in terms of total silver concentration and the results for ionic silver were expressed as percentages of total silver (AgNPs + Ag+). Samples for total silver determinations were prepared in triplicate by dilution with acidified (HNO_3_) water as necessary. In order to establish possible sources of bias from the filtration membrane, procedural blanks were run in parallel and the recovery of ionic silver (10 μg/L) from the filtration unit was assessed. No silver was detected in the procedural blanks and the average recovery of ionic silver was found to be 102.6 ± 3.8 %, showing absence of silver release/adsorption during filtration.

### Animals

Male CD-1(ICR) mice of 4–5 weeks were purchased from Charles River (Calco, Italy). They were acclimated to the environment for a week prior to the initiation of the study, with free access to water and a standard pellet diet *ad libitum*. The environmental conditions were set at a temperature of 22 ± 2 °C, relative humidity of 55 ± 10 % and a 12 h light/dark cycle.

### Experimental design

Mice were randomly assigned to groups of treatments. The mice were intravenously (IV) injected into the lateral tail vein with AgNPs of different sizes (10 nm, 40 nm, 100 nm), either CT- or PVP-coated, at a single dose of 10 mg/kg body weight (bw), and with AgAc at a single dose of 15.5 mg/kg, corresponding to 10 mg Ag/kg bw (3 mice per group). The AgNPs suspensions were administered to animals without any dilution. The control group was treated with sterile water for injection. Immediately after the treatment and the following hours, the general health and behavior of mice were monitored. The body weight of each mouse was measured before treatment and at sacrifice. Mice were euthanized 24 h after the treatment. The experiment was approved by an independent Ethical Committee on Animal Experimentation (Ethical Committee of the University of Milan, Opinion no. 81/14) and was performed in accordance with the Italian Laws (D.L.vo 116/92 and following additions), which enforce EU 86/609 Directive.

### Sampling

At 24 h after IV administration mice were euthanized by carbon dioxide inhalation. After drawing blood from the heart, mice underwent complete necropsy. Blood, liver, spleen, kidneys, lungs, and brain were collected for silver quantification and histopathological examination. The organ weight was measured and relative organ weights (%) were calculated as wet organ weight/total body weight. For quantification of silver, blood and a portion of the collected organs were stored at -80 °C for later analysis.

### Determination of silver

#### Chemicals

Ultrapure deionized water obtained by a Milli-Q Element System (Millipore, Molsheim, France), ultrapure grade nitric acid (67–69 % v/v) (Carlo Erba Reagenti, Rodano, Italy), ultrapure grade hydrochloric acid (32-35 % v/v) (Romil Ltd, Cambridge, UK) and ultrapure grade hydrogen peroxide (30 % v/v) (Merck, Darmstadt, Germany) were used throughout. For ICP-MS measurements, silver calibrants and rhodium solutions used were obtained from standard certified solutions of 1 mg/ml (High Purity Standard, Charleston, SC) by dilution with acidified (HNO3 and HCl) water, as necessary.

#### Determination of total silver content

Total silver concentrations were determined in organs and whole blood by means of a triple quadrupole inductively coupled plasma mass spectrometer (ICP-MS). A 8800 ICP-QQQ spectrometer (Agilent Technologies, Japan, Tokio) equipped with an autosampler, a peristaltic pump, a MicroMist glass concentric nebuliser, and operated at a RF power of 1550 W, was used. All sample manipulations were carried out in clean room conditions under a laminar flow box (Spetec GmbH, Erding, Germany). Before ICP-MS measurements, whole organs and blood were placed in Falcon tubes and pre-digested for 5 h at room temperature with 2–4 ml HNO_3_, depending on the organ weight. After adding 1 ml H_2_O_2_, samples were digested in a microwave system (Milestone Ethos E microwave labstation, FKV, Bergamo, Italy) at 90 °C for 8 h, maximum power 800 W. After cooling, the digests were diluted by adding HCl (final concentration 3.0 M) to promote the formation of soluble silver complexes and prevent the precipitation of insoluble Ag^+^ salts. Prior to analysis the digests were highly diluted with 0.1 % HNO_3_ and the appropriate amount of HCl so as to maintain the silver in complexed form. Measurements were carried out on ^107^Ag and ^103^Rh, as internal standard, by the method of external calibration. The method detection limit ranged from 0.4 to 0.7 μg/kg, depending on the tissue, and was 0.09 μg/l for blood.

#### Analytical quality control

Trueness of ICP-MS measurements was assessed by analysing the certified reference material SRM 1577c Bovine Liver (NIST, Gaithersburg, MD, USA), with a certified value for silver of 5.9 ± 1.6 μg/kg and the control material Seronorm™ Trace Elements Whole Blood L-1 (SERO AS, Billingstad, Norway) with a indicative value for silver of 185 ± 10 ng/l, both included in every analytical batch. The average determined silver concentrations were 6.0 ± 0.5 μg/kg (*n* = 6) and 179 ± 2 ng/l (*n* = 6) for the liver-based and the blood-based materials, respectively, in good agreement with the reference values. The trueness of determinations was also assessed through spikes of known amounts of silver in tissues and blood before sample dissolution, giving recoveries within the range of 90-100 % with no appreciable differences between sample types.

### Histopathological examination

For histological examination, liver (median lobe including the gall bladder), spleen (apical portion), kidney (half of the right kidney), lung (left lobe), brain (half brain, cut along the sagittal plane) were fixed in 10 % neutral buffered formalin for at least 48 h at room temperature, routinely processed for paraffin embedding, sectioned at 4 μm thickness, stained with hematoxylin-eosin (H&E), and evaluated under a light microscope. Grading of histopathological lesions in the examined organs was performed as follows: 0 = absence of lesions; 1 = minimal lesions; 2 = mild lesions; 3 = moderate lesions; 4 = severe lesions.

To analyze the tissue distribution and localization of silver, autometallography (AMG) [42] and immunofluorescence were performed on serial sections. After AMG staining, sections were counterstained with safranin O and evaluated under a light microscope for the identification of tissue and cellular localization of silver, visible as black granular pigment. For immunofluorescence, liver sections were immunostained with rabbit monoclonal anti-IBA1 antibody (Wako Chemicals Richmond, VA, USA, cat. No. 019-19741), a pan-macrophage marker [68]. Secondary antibody, Alexa Fluor® 555 F(ab’)2 Fragment of Goat Anti-Rabbit IgG (H + L) (Life Technologies Europe BV, Monza, Italy, cat. No. A-21430) was then added. Immunofluorescently labeled sections were acquired with the Leica TCS SP5 confocal microscope (Leica Microsystems GmbH, Wetzlar, Germany). The Alexa555 fluorophore was excited with the 561 nm laser line and the emitted fluorescence (570–700 nm) acquired with a 63x/1.4 oil immersion objective (Leica Microsystems GmbH). Nuclei were visualized by DAPI staining (405 nm laser line excitation, 415–500 nm acquisition window). Silver aggregates were visualized by reflection of light at 561 nm [24].

### Statistical analysis

Data were analyzed using Graph Pad Prism version 5.0 (GraphPad Software, San Diego, CA). For TEM, paired samples *t*-test was performed. Since the number of data (weights and silver concentrations) obtained from *in vivo* experiments was small, nonparametric tests (Kruskal-Wallis and Mann–Whitney *U* test) were used to detect differences between groups. The *P*-values <0.05 were considered statistically significant. Data were additionally explored by Principal Component Analysis, in order to understand the relationships among variables and their effect on data distribution.

## Additional file

Additional file 1: Table S1. Body weight gain and relative organ weight (%) after IV administration of differently coated AgNPs. **Table S2.** Silver tissue concentration determined by ICP-MS after IV administration of AgNPs and AgAc. **Figure S1.** Silver tissue concentration after IV administration of differently coated AgNPs and AgAc. **Figure S2.** Principal component analysis of silver tissue concentration data. **Figure S3.** Histology of spleen and liver after IV administration of 10 mg Ag/Kg in mice. **Figure S4.** Histology of liver from mice treated with 10 nm AgNPs. (PDF 728 kb)

## Abbreviations

AgAc: silver acetate
AgNP: silver nanoparticle
AMG: autometallography
bw: body weight
CT: sodium citrate
DLS: dynamic light scattering
H&E: hematoxylin and eosin
ICP-MS: inductively coupled plasma mass spectroscopy
PVP: polyvinylpyrrolidone
TEM: transmission electron microscopy
UV–Vis: UV-visible
