# Supplementary material for: Tissue distribution and acute toxicity of silver after single intravenous administration in mice: nano-specific and size-dependent effects
Source: Part Fibre Toxicol. 2016 Feb 29;13:12. doi: 10.1186/s12989-016-0124-x (PMC4772516; doi:10.1186/s12989-016-0124-x)
Supplement: Additional file 1: Table S1. — Body weight gain and relative organ weight (%) after IV administration of differently coated AgNPs. Table S2. Silver tissue concentration determined by ICP-MS after IV administration of AgNPs and AgAc. Figure S1. Silver tissue concentration after IV administration of differently coated AgNPs and AgAc. Figure S2. Principal component analysis of silver tissue concentration data. Figure S3. Histology of spleen and liver after IV administration of 10 mg Ag/Kg in mice. Figure S4. Histology of liver from mice treated with 10 nm AgNPs. (PDF 728 kb) [file 12989_2016_124_MOESM1_ESM.pdf]

Supplementary Material

Tissue distribution and acute toxicity of silver after single intravenous administration in mice: nano-specific and size-dependent effects

Camilla Recordati[1, ‡,*], Marcella De Maglie[1,2, ‡], Silvia Bianchessi[1], Simona Argentiere[1], Claudia Cella[1,3], Silvana Mattiello[2], Francesco Cubadda[4], Federica Aureli[4], Marilena D'Amato[4], Andrea Raggi[4], Cristina Lenardi[1,3,5], Paolo Milani[1,3,5], Eugenio Scanziani[1,2]

[1]Fondazione Filarete, 20139 Milano, Italy

[2]Dipartimento di Scienze Veterinarie e Sanità Pubblica (DIVET) - Università degli Studi di Milano, 20133 Milano, Italy,

[3]Dipartimento di Fisica - Università degli Studi di Milano, 20133 Milano, Italy,

[4]Department of Food Safety and Veterinary Public Health, Istituto Superiore di Sanità - National Health Institute, 00161 Rome, Italy

[5]Centro Interdisciplinare Materiali e Interfacce Nanostrutturati (CIMAINA) - Università degli Studi di Milano, 20133 Milano, Italy

‡These authors contributed equally.

*Corresponding author: Camilla Recordati camilla.recordati@fondazionefilarete.com.

Table S1. **Body weight gain and relative organ weight (%) after IV administration of differently coated AgNPs**. Data are expressed as means ± SD.

Group	n	Body weight gain	Spleen	Liver	Lung	Kidney	Brain
Control	3	-3.3 ± 5	0.45 ± 0.1	6.61± 0.8	0.66 ± 0.0	1.80 ± 0.2	1.77 ± 0.1
10 nm AgNP-CT	3	-14.8± 0.6	0.63 ± 0.3	7.86 ± 1.6	0.74 ± 0.2	1.75 ± 0.2	1.91 ± 0.2
10 nm AgNP-PVP	3	-9.3 ± 4.4	0.66 ± 0.3	6.60 ± 0.8	0.71 ± 0.1	1.71 ± 0.1	1.71 ± 0.2
40 nm AgNP-CT	3	0.0 ± 3.0	0.43 ± 0.1	6.53 ± 0.6	0.65 ± 0.2	1.64 ± 0.2	1.73 ± 0.1
40 nm AgNP-PVP	3	-2.6 ± 2.3	0.45 ± 0.1	7.21 ± 0.6	0.66 ± 0.0	1.95 ± 0.3	1.83 ± 0.1
100 nm AgNP-CT	3	-1.4 ± 1.6	0.39 ± 0.0	7.01 ± 0.3	0.61 ± 0.1	1.86 ± 0.2	1.66 ± 0.1
100 nm AgNP-PVP	3	1.2 ± 7,5	0.40 ± 0.1	6.92 ± 0.2	0.65 ± 0.0	1.73 ± 0.0	1.64 ± 0.1
AgAc	3	-6.3± 3.3	0.47± 0.0	6.99± 0.0	0.80 ± 0.1	1.92± 0.2	1.78 ± 0.2

Table S2. Silver tissue concentration determined by ICP-MS after IV administration of AgNPs and AgAc. Data are expressed as means (µg/g wet organ).

Group	Mouse ID	Organ					
		Spleen	Liver	Lung	Kidney	Brain	Blood
Control	B1	0.002	0.004	0.039	0.005	0.001	0.000
	C2	0.006	0.005	0.017	0.002	0.001	0.000
	A3	0.002	0.002	0.003	0.002	0.001	n.a.
	**Mean**	**0.003**	**0.004**	**0.020**	**0.003**	**0.001**	**0.000**
	SD	0.002	0.001	0.018	0.001	0.000	0.000
10 nm AgNP-CT	A1	113.6	77.6	73.6	2.2	0.4	2.7
	B2	112.4	71.3	62.9	3.3	0.3	n.a.
	C3	139.1	85.7	66.1	4.0	0.3	2.4
	**Mean**	**121.7**	**78.2**	**67.5**	**3.2**	**0.3**	**2.6**
	SD	15.1	7.2	5.5	0.9	0.1	0.3
10 nm AgNP-PVP	F1	81.6	63.8	34.8	3.0	0.2	n.a.
	G2	100.0	69.5	32.1	2.5	0.4	2.2
	H3	82.3	74.2	31.3	2.9	0.3	2.1
	**Mean**	**88.0**	**69.2**	**32.8**	**2.8**	**0.3**	**2.2**
	SD	10.5	5.2	1.8	0.3	0.1	0.1
40 nm AgNP-CT	E5	70.9	62.9	8.4	0.5	0.2	1.5
	G3	109.7	79.3	10.1	1.0	0.2	1.1
	H1	72.0	70.8	5.6	0.9	0.2	2.7
	**Mean**	**84.2**	**71.0**	**8.0**	**0.8**	**0.2**	**1.8**
	SD	22.1	8.2	2.3	0.3	0.0	0.8
40 nm AgNP-PVP	A5	80.9	69.3	6.0	1.2	0.2	1.3
	A6	68.6	67.7	4.9	0.9	0.2	0.8
	E1	59.6	58.2	4.7	0.9	0.2	1.0
	**Mean**	**69.7**	**65.0**	**5.2**	**1.0**	**0.2**	**1.0**
	SD	10.7	6.0	0.7	0.2	0.0	0.2
100 nm AgNP-CT	C5	67.6	51.4	7.6	0.4	0.2	0.8
	E6	61.9	56.9	10.9	0.5	0.2	0.7
	D4	78.2	51.5	5.9	0.6	0.2	0.7
	**Mean**	**69.2**	**53.2**	**8.1**	**0.5**	**0.2**	**0.7**
	SD	8.3	3.1	2.5	0.1	0.0	0.0
100 nm AgNP-PVP	D3	60.5	61.6	1.9	0.9	0.2	0.6
	F4	54.8	62.7	2.8	0.7	0.2	0.7
	G5	68.3	71.9	2.9	0.6	0.2	0.6
	**Mean**	**61.2**	**65.4**	**2.5**	**0.7**	**0.2**	**0.6**
	SD	6.8	5.7	0.5	0.1	0.0	0.0
AgAc	H4	11.8	55.2	3.3	16.5	0.36	n.a.

H5	19.1	63.2	4.1	13.8	0.43	n.a.
H6	10.6	78.6	4.3	10.2	0.36	n.a.
**Mean**	**13.9**	**65.7**	**3.9**	**13.5**	**0.38**	**n.a.**
SD	4.6	11.9	0.5	3.2	0.04	n.a.

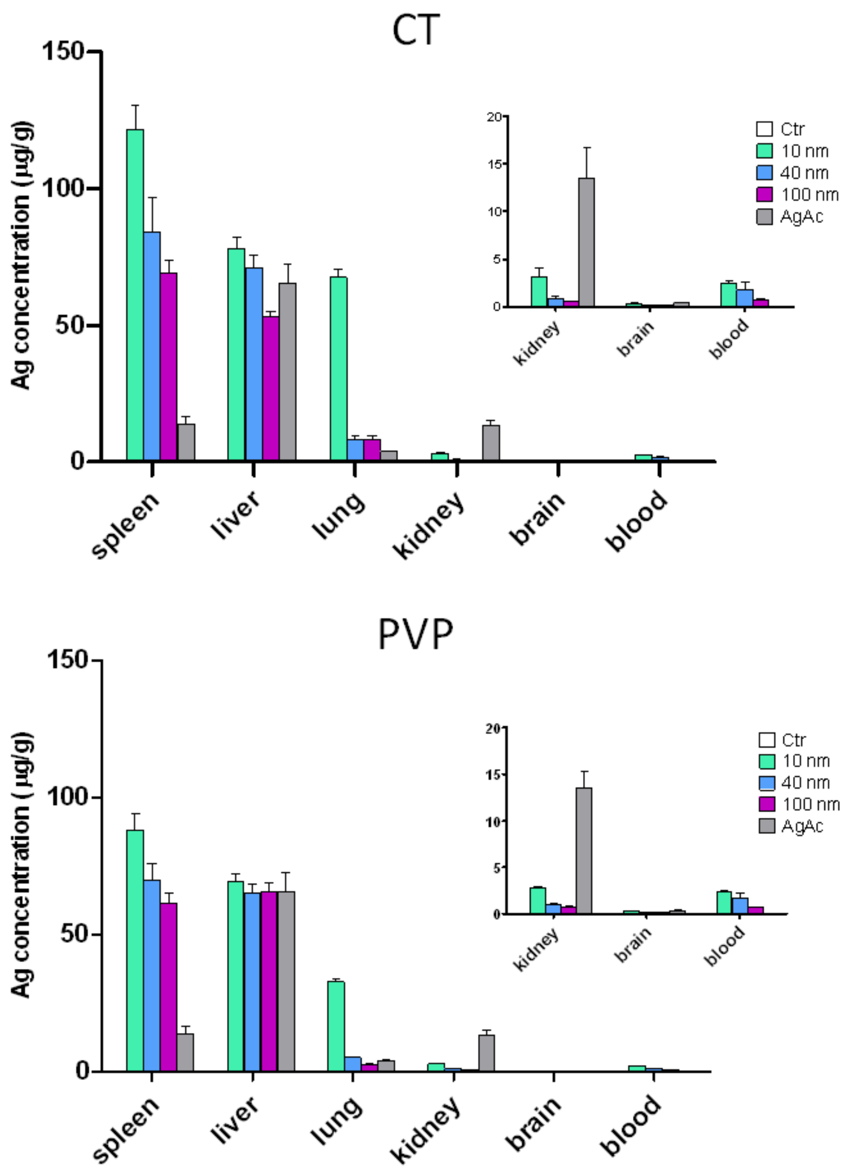

Fig. S1. Silver tissue concentration after IV administration of differently coated AgNPs and AgAc. Data are expressed as means ± SD.

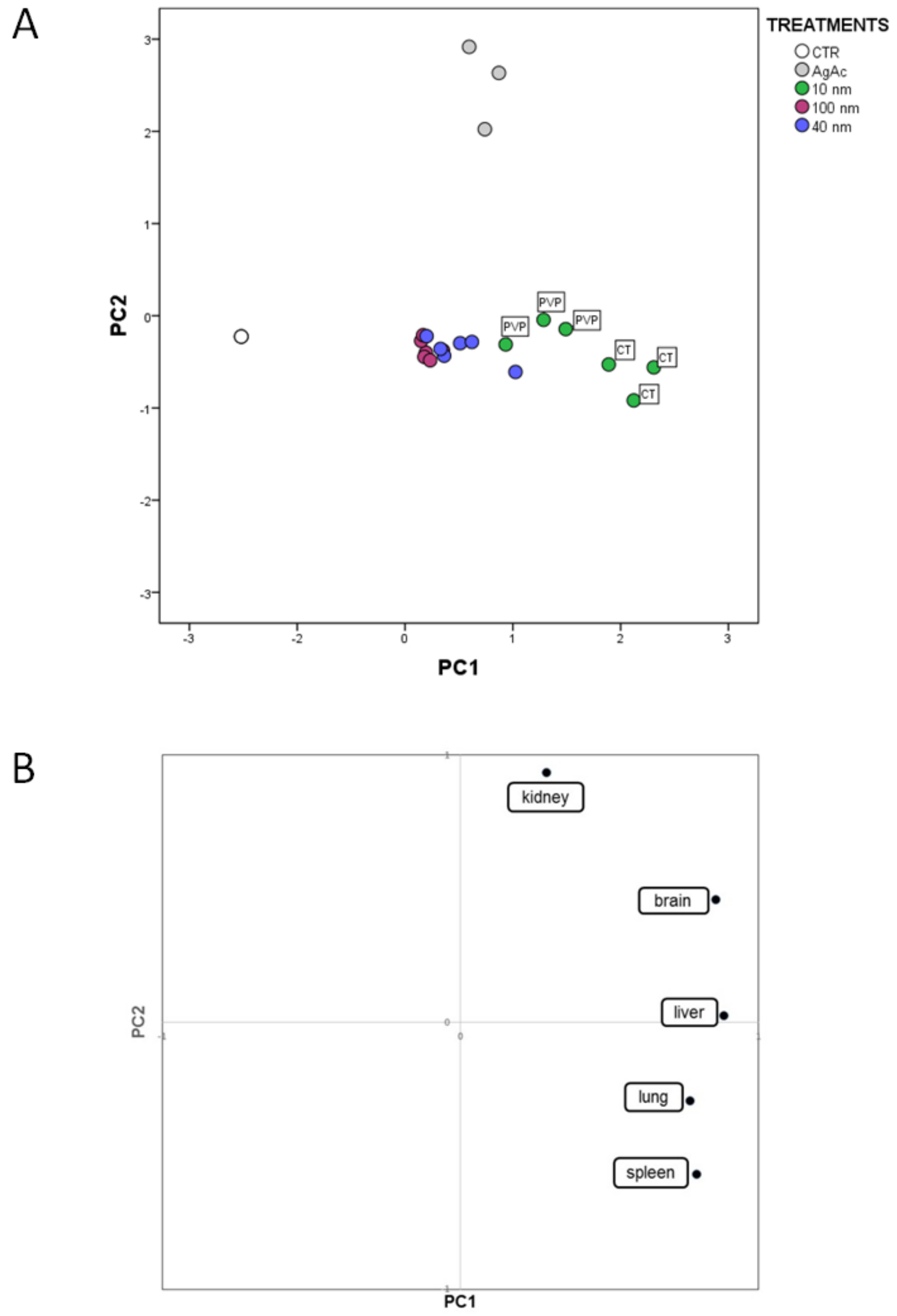

Fig. S2. Principal component analysis of silver tissue concentration data. PCA score plot (A) and loading plot (B) on the first two Principal Components (PCs), which explain 86.6% of the total variance (explained variance of 56.7% and 29.8% for PC1 and PC2, respectively). The score plot represents data distribution, whereas the loading plot shows the relative weight of each

variable (silver concentration in the considered organs) on the first two PCs. Data are classed by treatment (Control, 10 nm AgNPs, 40 nm AgNPs, 100 nm AgNPs, and AgAc). The effect of coating (CT or PVP) is highlighted only for AgNPs 10 nm, where this effect was visible, whereas for the other treatments data distribution did not allow to separate CT from PVP.

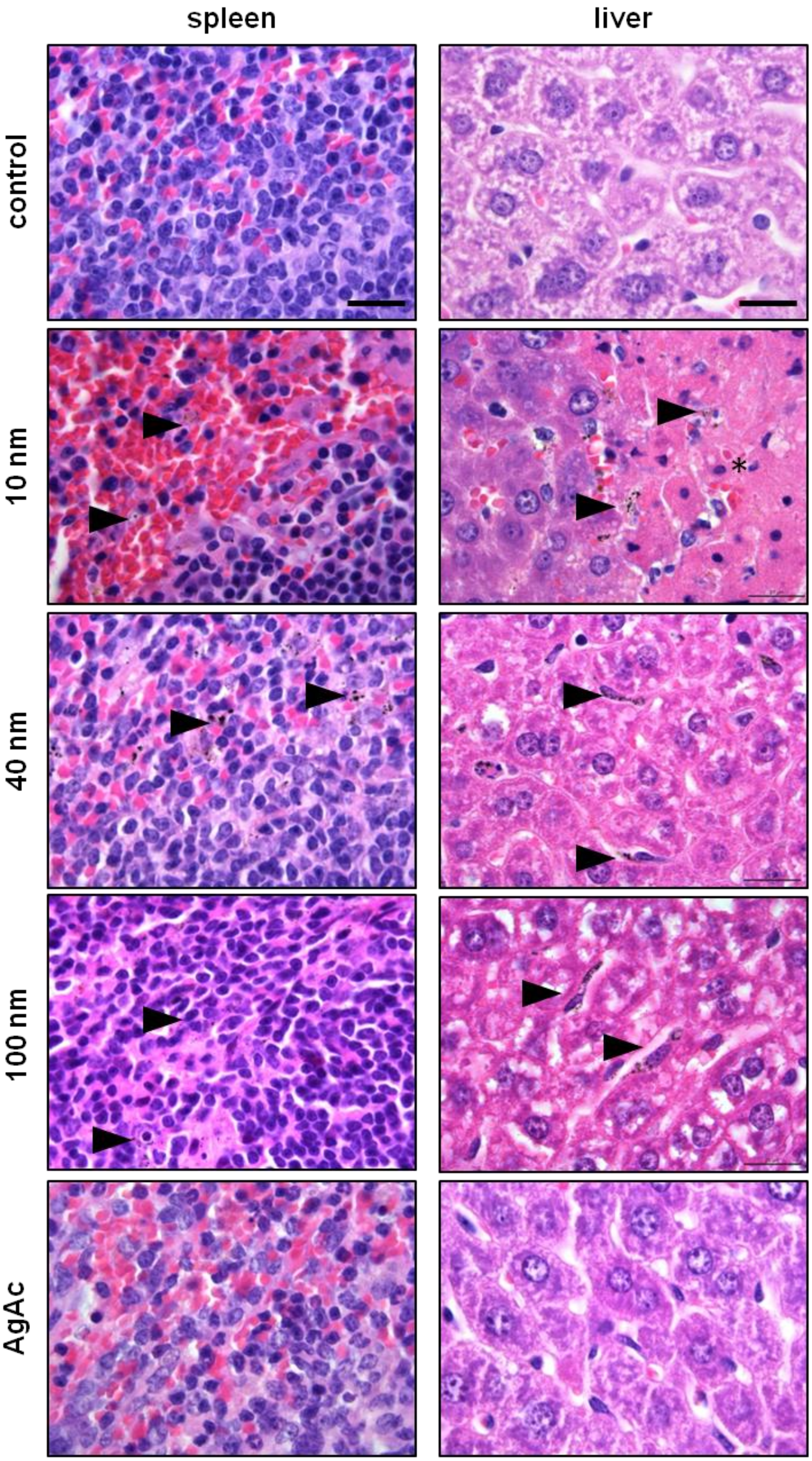
spleen
liver
control
10 nm
40 nm
100 nm
AgAc

Fig. S3. Histology of spleen and liver after IV administration of 10 mg Ag/Kg in mice. Representative images of spleen and liver (scale bar = 20 μm) from vehicle- (control), AgNP (10 nm, 40 nm, 100 nm), and AgAc-treated mice. In H&E stained sections intracytoplasmic black granular pigment (arrowheads) was multifocally (and often barely) visible in the spleen (marginal zone and red pulp) and liver (along the sinusoids) of all AgNP-treated mice, but not in AgAc-treated mice. In 10 nm AgNP-treated mice, black granular material was also evident in the areas of hepatic necrosis (*) (scale bar = 20 μm).

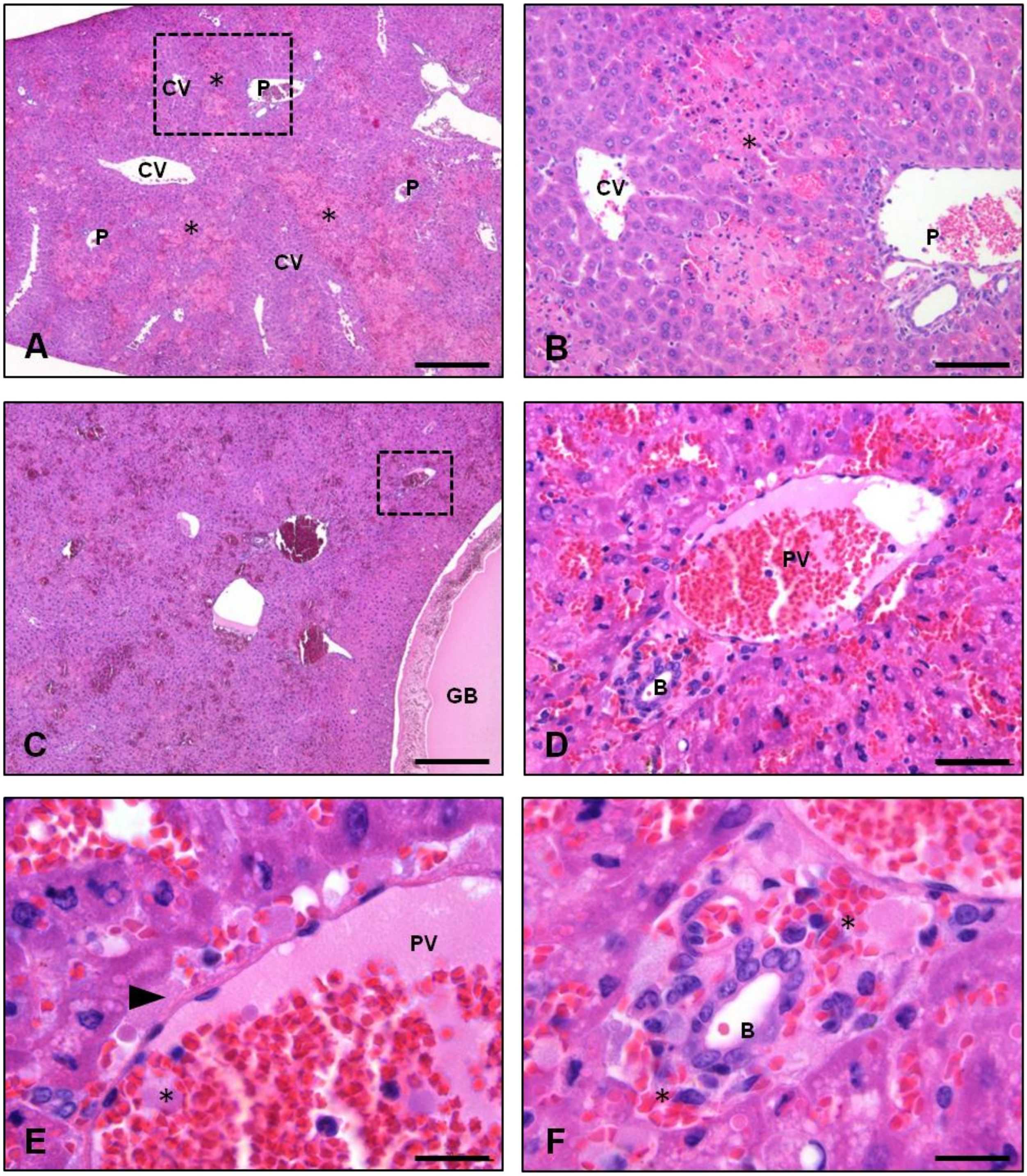
A
CV
*
P
CV
P
P
*
*
CV
B
CV
*
P
C
GB
D
PV
B
E
PV
*
F
*
B
*

Fig. S4. Histology of liver from mice treated with 10 nm AgNPs. A-B) Characteristic pattern of midzonal hepatocellular necrosis (*), affecting the intermediate region of hepatic lobule between the portal tracts (P) and centrilobular veins (CV) (A, scale bar = 400 µm; B, scale bar = 100 µm). C-F) In this liver, in addition to the midzonal hepatocellular necrosis, portal to midzonal hemorrhages were particularly prominent, as well as the gall bladder (GB) mural hemorrhage. At level of the portal tracts, endothelial damage of the portal vein (PV) with presence of subendothelial hemorrhages (arrowhead), and intraluminal fibrin thrombi (*) was found. Peribiliary microhemorrhages (*) were also evident (B = bile duct) (C, scale bar = 400 µm; D, scale bar = 50 µm; E-F, scale bar = 20 µm) (H&E staining**).**
